# Anti-osteoarthritic Effects of an Herbal Composition LI73014F2 on Interleukin-1β-induced Primary Human Articular Chondrocytes

**DOI:** 10.3390/molecules25092033

**Published:** 2020-04-27

**Authors:** Hae Lim Kim, Hae Jin Lee, Dong-Ryung Lee, Bong-Keun Choi, Seung Hwan Yang

**Affiliations:** 1 Department of Biotechnology, Chonnam National University, Yeosu 59626, Korea; ics1357@naver.com (H.L.K.); haecutejin@naver.com (H.J.L.); 2 Nutrapharm Tech, Jungwon-gu, Seongnam, Gyunggi 13201, Korea; drlee@nutrapharm.co.kr (D.-R.L.); cbcbcbk@nutrapharm.co.kr (B.-K.C.)

**Keywords:** anti-inflammation, apoptosis, *Boswellia serrata* extracts, *Terminalia chebula* fruit extracts, *Curcuma longa* rhizome extracts, human articular chondrocyte, interleukin-1β, LI73014F2, metalloproteinases

## Abstract

Osteoarthritis (OA) is one of the most well-characterized joint diseases and is associated with chondrocyte inflammation, metalloproteinase upregulation and apoptosis. LI73014F2 is a novel composition prepared from aqueous extract of *Terminalia chebula* fruit, alcohol extract of *Curcuma longa* rhizome, and *Boswellia serrata* extract at 2:1:2 ratio. Earlier studies have shown that LI73014F2 inhibits cyclooxygenase-2 (COX-2), 5-lipoxygenase (5-LOX) activities, and attenuates clinical symptoms in OA subjects. In the present study, we evaluated the protective anti-inflammatory and anti-apoptotic effects, as well as the underlying mechanisms, of LI73014F2 in interleukin (IL)-1β-induced inflammation in human primary chondrocytes. Human chondrocytes were treated with LI73014F2 (0, 12.5, 25 and 50 μg/mL) in IL-1β (10 ng/mL)-containing chondrocyte growth medium for 24 h. Cell viability was assessed using an MTT assay. The pro-inflammatory mediator, inflammatory cytokines, MMPs, apoptosis-related proteins, mitogen-activated protein kinase (MAPK) and nuclear factor-κB (NF-κB) signaling pathways protein expression levels were detected by western blot analysis. The results demonstrated that LI73014F2 normalized the expressions of COX-2, mPGES-1, PGE_2_, 5-LOX, LTB_4_, IL-1β, TNFα, IL-6, MMP-2, MMP-3, MMP-9, MMP-13, Bax/Bcl-2, cleaved caspase-9 and -3, cleaved PARP, phospho-NF-κB p65 and phospho-p38 MAPK proteins in IL-1β-induced primary human chondrocytes. Moreover, the data suggested that LI73014F2 reduced IL-1β-induced inflammation and apoptosis, at least partially via the inhibition of the NF-κB/MAPK signaling pathway. In conclusion, the present findings provide the molecular basis of the anti-OA efficacy of LI73014F2.

## 1. Introduction

Osteoarthritis (OA) is a degenerative joint disease characterized by abnormal changes in the structure, composition, and function of joint tissues and affects tens of millions of individuals worldwide [1,2]. Chondrocytes are the main cell type in cartilage and are responsible for the synthesis and conversion of the extracellular matrix, which is crucial for joint function [3]. The overproduction of pro-inflammatory cytokines, such as IL-1β and tumor factor necrosis α (TNFα), is involved in the pathogenesis of OA by up-regulating metalloproteinases (MMPs) and inducing apoptosis [4]. Interleukin-1β (IL-1β) is a pro-inflammatory cytokine that plays a critical role in the development of OA [5]. Treatment with IL-1β stimulated the release of cyclooxygenase-2 (COX-2) and 5-lipoxygenase (5-LOX) in chondrocytes, leading to the production of prostaglandin E_2_ and LTB_4_, respectively [6,7,8]. Therefore, there are reasons in many studies to explained that IL-1β production and IL-1β-induced inflammatory mediators plays a major role the progression of OA [9].

Previous research has also shown that IL-1β may activate nuclear factor (NF)-κB and mitogen-activated protein kinase (MAPK), which regulate the expression of several other proinflammatory cytokines and proteases and mediate critical events such as apoptosis in the inflammatory responses of chondrocytes [10,11,12].

Extracts of *Terminalia chebula*, *Curcuma longa* and *Boswellia serrata* have long been used as traditional Ayurvedic medicines for the treatment of several types of inflammatory diseases [13,14,15]. Previous studies have demonstrated that various forms of *B. serrata* extracts are safe for consumption and are effective in alleviating the clinical symptoms of OA [16,17,18,19]. *C. longa* extracts are potent anti-inflammatory and antioxidant agents that have demonstrated an excellent safety profile and clinical efficacy in several diseases including knee osteoarthritis [20,21]. Furthermore, preclinical and clinical evaluations revealed that *T. chebula* extracts exhibit disease-modifying activities in OA [22,23].

LI73014F2 is a novel synergistic composition comprising the extracts of *T. chebula* fruit, *C. longa* rhizome and *B. serrata* gum resin. The results from previous clinical study [24] showed that LI73014F2 inhibited COX-2 and 5-LOX enzymatic activity more than when treated with the individual ingredients. Therefore, based on the results from previous clinical study, we investigated whether LI73014F2 treatment can further reduced PGE_2_ and LTB_4_ levels than the standalone individual extract treatment. Furthermore, we investigated the effects of LI73014F2 on IL-1β-stimulated inflammation in human chondrocytes and evaluated the expression of MMPs and apoptotic effects, to elucidate the underlying p38 MAPK and NF-κB p65 signaling pathway.

## 2. Materials and Methods

### 2.1. Preparation of Individual Extracts and LI73014F2

LI73014F2 is mixture consisting of three materials. It is a synergistic composition comprising the aqueous extract of *T. chebula* fruit, alcoholic extract of *C. longa* rhizome, and *B. serrata* extract in a ratio of 2:1:2 and LI73014F2 was prepared identically to the previously reported study [24]. Individual extracts of *T. chebula* (TCE, LI73000), *C. longa* (CLE, LI01106) and *B. serrata* (BSE, LI13121), were obtained from Laila Nutraceuticals. LI73014F2 was also obtained from Laila Nutraceuticals. For in vitro studies individual extracts or LI73014F2 were dissolved in dimethyl sulfoxide (DMSO) at concentration of 50 mg/mL and then diluted in chondrocyte growth media at concentration of 50 μg/mL, respectively.

### 2.2. Chemicals and Reagents

Chondrocyte growth media was purchased from PromoCell Bioscience Alive (Heidelberg, Germany). Primary antibodies against β-actin, COX-2, Bax, Bcl-2, cleaved caspase-9 and -3, cleaved poly (ADP-ribose) polymerase (PARP), nuclear factor (NF)-κB p65, phospho-NF-κB p65, phospho-p38 mitogen-activated protein kinase (MAPK), and p38 MAPK were purchased from Cell Signaling Technology (Danvers, MA, USA). Microsomal PGE_2_ synthase-1 (mPGES-1), prostaglandin E_2_ (PGE_2_), 5-LOX, interleukin (IL)-1β, tumor necrosis factor (TNF)α, IL-6, matrix metalloproteinase (MMP)-2, MMP-3, MMP-9, and MMP-13 were obtained from Abcam (Cambridge, MA, USA). Leukotriene B_4_ (LTB_4_) was purchased from Enzo Life Sciences (Farmingdale, NY, USA). Horseradish peroxidase (HRP)-conjugated anti-rabbit and anti-mouse immunoglobulin G (IgG) secondary antibodies were purchased from GenDEPOT (Barker, TX, USA). IL-1β was obtained from PeproTech, Inc. (Rocky Hill, NJ, USA). Dimethyl sulfoxide was obtained from Daejung Chemicals & Metals Co., Ltd. (Siheung, Korea).

### 2.3. Culture and Treatment of Human Articular Chondrocytes

Human articular chondrocytes (HCHs) were purchased from PromoCell Bioscience Alive (Heidelberg, Germany) and maintained in complete chondrocyte growth medium (PromoCell, Heidelberg, Germany) supplemented with 10% fetal calf serum in a humidified incubator at 37 °C and 5% CO_2_. Upon reaching 80% confluence, the HCH cells were passaged, and cells at P1 were used for subsequent experiments. HCH cells seeded at a density of 1 × 10^5^ cells/well in a 6-well plate were treated with increasing concentrations (0, 12.5, 25 and 50 μg/mL) of LI73014F2 in the presence of IL-1β (10 ng/mL) for 24 h. Vehicle control is HCH cells treated with growth media containing only DMSO (final DMSO concentration 0.1%).

### 2.4. Cell Viability Assay

Cell viability was determined by the 3-[4, 5-dimethylthiazol-2-yl]-2, 5 diphenyl tetrazolium bromide (MTT) assay. HCH cells were treated with increasing concentrations of LI73014F2 (0, 12.5, 25 and 50 μg/mL) for 24 h. MTT solution (5 mg/mL) was added to each of the wells, and the cells were incubated for 3 h at 37 °C. The supernatant was removed from each well, and DMSO was added to dissolve the formazan crystals. The absorbance was measured at a wavelength of 570 nm using a microplate reader (Tecan, Männedorf, Switzerland).

### 2.5. Protein Extraction and Western Blot Analysis

HCH cells were lysed in CelLytic buffer (Sigma-Aldrich, MO, USA) and the lysates were centrifuged at 12,000 rpm for 15 min at 4 °C. The protein concentrations were determined by a Bradford assay (Bio-Rad Laboratories, Hercules, CA, USA). The proteins were separated via SDS-PAGE on 10% gels and transferred onto Immobilon-P membranes (Millipore, Bedford, MA, USA). The membranes were blocked with 5% skimmed milk in Tris-buffered saline containing 0.1% Tween-20 for 1 h, and then incubated with specific primary antibodies against β-actin, COX-2, mPGES-1, PGE_2_, 5-LOX, LTB_4_, IL-1β, TNFα, IL-6, MMP-2, MMP-3, MMP-9, MMP-13, Bax, Bcl-2, cleaved caspase-9 and -3, cleaved PARP, phospho-NF-κB p65, NF-κB p65, phospho-p38 MAPK and p38 MAPK (dilution 1:1000) overnight at 4 °C. Following primary antibody incubation, the membranes were incubated with the corresponding HRP-conjugated anti-rabbit and anti-mouse IgG secondary antibodies (dilution 1:10,000) for 1 h at 23 °C. The bands were visualized with ECL solution (GenDEPOT, Barker, TX, USA) and the intensity of bands was detected using a LuminoGraph chemiluminescent imaging system (Atto, Tokyo, Japan). β-actin was used as a control for normalization. western blot bands were analyzed using Image J software.

### 2.6. Statistical Analysis

All data are presented as the mean ± standard deviation. Groups were compared using the Student’s t-test and one-way analysis of variance, as applicable. Statistical analyses were performed using Origin 7 software (Microcal Software, Northampton, MA, USA). *P* < 0.05 was considered to indicate a statistical significance

## 3. Results

### 3.1. Effect of LI73014F2 on the Viability of HCH Cells

**Figure 1:**
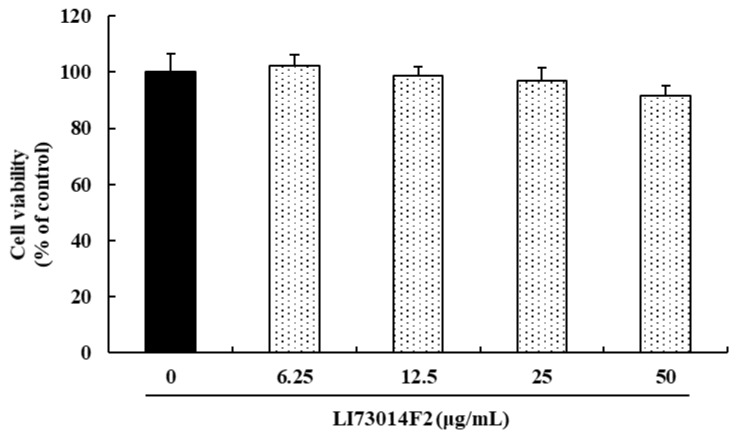
Effect of LI73014F2 on cell viability in Human articular chondrocytes (HCH cells). HCH cells were treated with increasing concentrations of LI73014F2 (0, 12.5, 25 and 50 μg/mL) for 24 h. The cell viability was evaluated using the 3-[4, 5-dimethylthiazol-2-yl]-2, 5 diphenyl tetrazolium bromide (MTT) assay.

The impact of LI73014F2 on the viability of HCH cells were evaluated using the MTT assay. LI73014F2 did not exhibit cytotoxic activity at any of the concentrations investigated (0, 12.5, 25 and 50 μg/mL) (Figure 1).

### 3.2. LI73014F2 Suppressed the IL-1β-induced Expression of COX-2, mPGES-1, PGE_2_, 5-LOX and LTB_4_ in HCH Cells

The previous study has shown the LI73014F2 is a synergistic composition comprising aqueous extract of *T. chebula* fruit, alcohol extract of *C. longa* rhizome and *B. serrata* extract at 2:1:2 ratio exhibited the best inhibitory enzymatic activity of COX-2 and 5-LOX in spectrophotometric assays (data not shown) [24].

Based on the previous clinical study [24], this study demonstrated the synergistic inhibitory effect of LI73014F2 on COX-2 and 5-LOX metabolic pathways products, such as PGE_2_ and LTB_4_.

**Figure 2:**
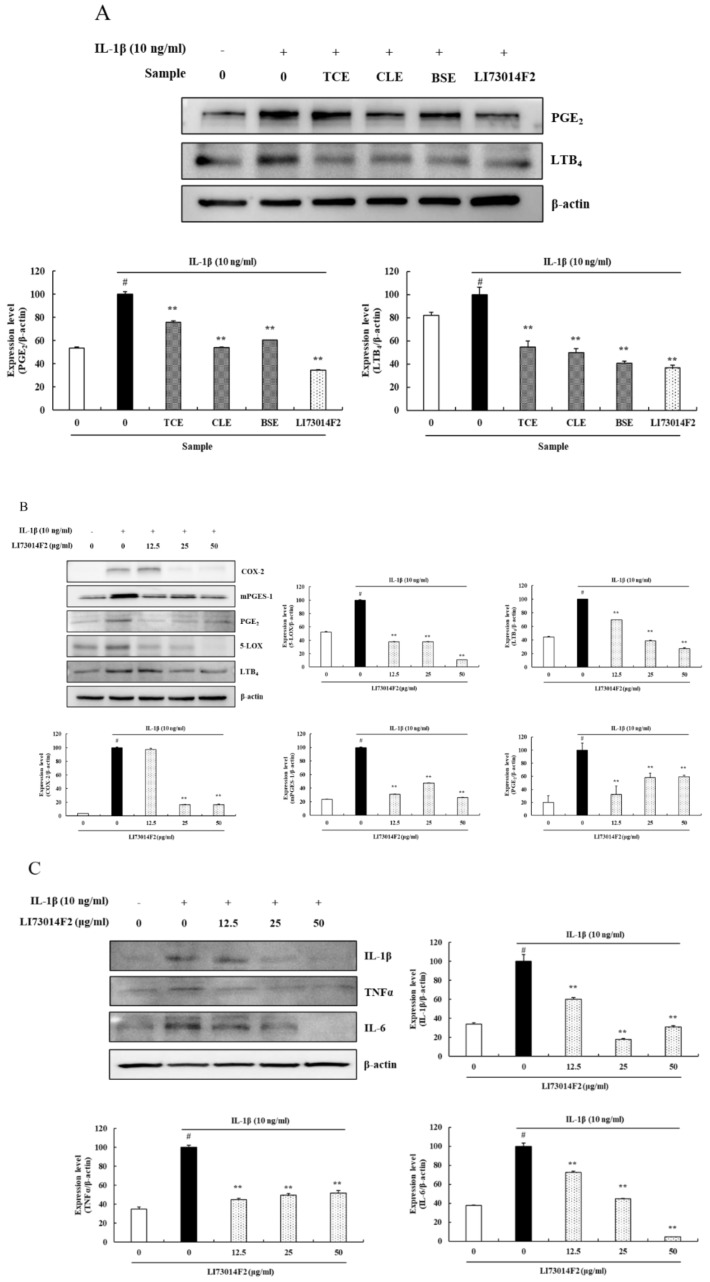
Effect of LI73014F2 on the expression levels of COX-2, mPGES-1, PGE_2_, 5-LOX, LTB_4_, IL-1β, TNFα and IL-6 in IL-1β-stimulated HCH cells. HCH cells were treated with IL-1β (10 ng/mL) alone or in combination with extracts of *T. chebula* fruit (TCE) or extracts of *C. longa* rhizome (CLE) or extracts of *B. serrata* (BSE) or LI73014F2 for 24 h. (**A**). Effects of LI73014F2 on the expression of PGE_2_ (42 kDa) and LTB_4_ (36 kDa) compared with those of its individual extracts. (**B**) Effects of LI73014F2 on the expression of COX-2 (74 kDa), mPGES-1 (17 kDa), PGE_2_, 5-LOX (78 kDa) and LTB_4_ in IL-1β-stimulated HCH cells. (**C**) Effects of LI73014F2 on the expression of IL-1β (17 kDa), TNFα (26 kDa) and IL-6 (25 kDa) in IL-1β-stimulated HCH cells. Western blot bands were analyzed using Image J software. β-actin was used as a control for normalization. # *P* < 0.05, compared with the vehicle control group. * *P* < 0.05 and ** *P* < 0.01, compared with the IL-1β-treated control group.

HCH cells in different groups were treated with individual extracts of *T. chebula* fruit (TCE; 50 μg/mL) or extracts of *C. longa* rhizome (CLE; 50 μg/mL) or extracts of *B. serrata* (BSE; 50 μg/mL), respectively, while another group was treated with LI73014F2 (50 μg/mL). All the cells were maintained in IL-1β (10 ng/mL)-containing chondrocyte growth medium for 24 h. The protein expression levels of PGE_2_ and LTB_4_ were investigated by western blot analysis. The results showed that the PGE_2_ inhibitory effects of TCE, CLE, BSE and LI73014F2 were 24.21%, 46.10%, 39.58% and 65.64%, respectively (*P* < 0.01) and the LTB_4_ inhibitory effects of TCE, CLE, BSE and LI73014F2 were 45.32%, 50.30%, 59.34% and 63.40%, respectively (*P* < 0.01) (Figure 2A). Therefore, LI73014F2 significantly reduced the expression of PGE_2_ and LTB_4_ compared with those of its individual extracts. Moreover, to investigate the anti-osteoarthritic effects of LI73014F2, HCH cells were treated with increasing concentrations of LI73014F2 (0, 12.5, 25 and 50 μg/mL) in the presence of IL-1β (10 ng/mL) for 24 h. The protein expression levels of COX-2, mPGES-1, PGE_2_, 5-LOX and LTB_4_ were investigated by western blot analysis. The results indicated that LI73014F2 significantly downregulated the IL-1β-stimulated expressions of COX-2, mPGES-1, PGE_2_, 5-LOX and LTB_4_ in the range of 83.39–83.53% (*P* < 0.01), 52.52–73.79% (*P* < 0.01), 40.73–67.87% (*P* < 0.01), 62.49–89.30% (*P* < 0.01) and 30.32–72.76% (*P* < 0.01), respectively, compared with HCH cells stimulated with IL-1β (Figure 2B).

### 3.3. LI73014F2 Suppressed the IL-1β-induced Expression of Inflammatory Cytokines in HCH Cells

The effects of LI73014F2 on the protein expression of inflammatory cytokines were evaluated by treating HCH cells with increasing concentrations of LI73014F2 in IL-1β-containing chondrocyte growth medium for 24 h, as aforementioned. The protein expression levels of IL-1β, TNFα and IL-6 were examined using western blot analysis. The results demonstrated that IL-1β significantly induced the protein expression of IL-1β, TNFα and IL-6 compared with the vehicle control. However, the protein expression level of LI73014F2-treated HCH cells decreased in the range of 39.80–82.12% for IL-1β, 48.37–55.11% for TNFα and 27.75–95.36% for IL-6 compared with HCH cells stimulated with IL-1β (*P* < 0.01) (Figure 2C).

### 3.4. LI73014F2 Decreased the Production of MMP-2, MMP-3, MMP-9 and MMP-13 in IL-1β-stimulated HCH Cells

**Figure 3:**
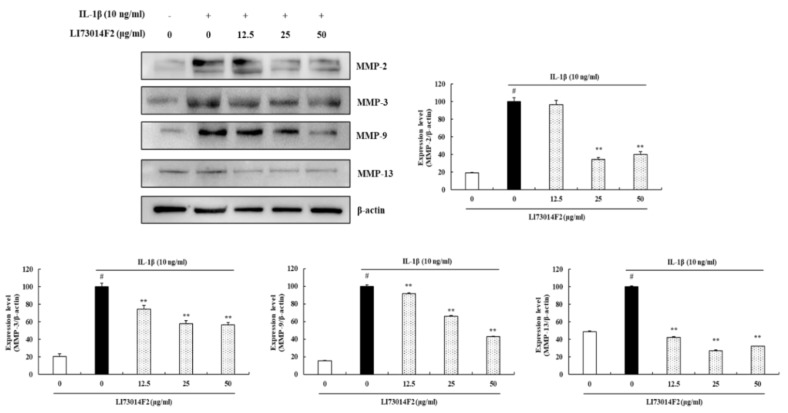
LI73014F2 decreased the production of MMP-2, MMP-3, MMP-9 and MMP-13 in IL-1β-stimulated HCH cells. HCH cells were treated with IL-1β (10 ng/mL) alone or in combination with LI73014F2 (0, 12.5, 25 and 50 μg/mL) for 24 h. The expression levels of MMP-2 (62, 72 kDa), MMP-3 (54 kDa), MMP-9 (92 kDa) and MMP-13 (54 kDa) were determined by western blot analysis. western blot bands were analyzed using Image J software. β-actin was used as a control for normalization. # *P* < 0.05, compared with the vehicle control group. * *P* < 0.05 and ** *P* < 0.01, compared with the IL-1β-treated control group.

As MMPs play critical roles in cartilage degradation, we investigated the effects of LI73014F2 on MMP-2, MMP-3, MMP-9 and MMP-13 expression in IL-1β-induced HCH cells using western blot analysis. The results of western blot analysis demonstrated that IL-1β significantly induced the protein expression of MMP-2, MMP-3, MMP-9 and MMP-13 compared with that in untreated HCH cells. Treatment with LI73014F2 for 24 h significantly reduced protein expression in the range of 59.95–65.78% for MMP-2 (*P* < 0.01), 25.59–43.38% for MMP-3 (*P* < 0.01), 8.3–57.24% for MMP-9 (*P* < 0.01) and 57.79–72.73% for MMP-13 (*P* < 0.01) compared with that in HCH cells stimulated with IL-1β (Figure 3).

### 3.5. Effects of LI73014F2 on IL-1β-induced Apoptosis in HCH Cells

**Figure 4:**
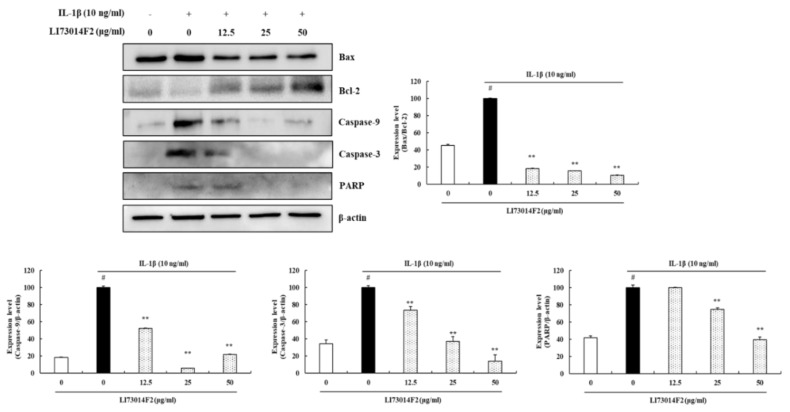
The effects of LI73014F2 on IL-1β-induced HCH cells apoptosis. HCH cells were treated with IL-1β (10 ng/mL) alone or in combination with LI73014F2 (0, 12.5, 25 and 50 μg/mL) for 24 h. The expression levels of Bax (20 kDa), Bcl-2 (26 kDa), cleaved caspase-9 (35 kDa) and -3 (17, 19 kDa) and cleaved PARP (89 kDa) were determined by western blot analysis. western blot bands were analyzed using Image J software. β-actin was used as a control for normalization. # *P* < 0.05, compared with the vehicle control group. * *P* < 0.05 and ** *P* < 0.01, compared with the IL-1β-treated control group.

To explore the mechanism by which LI73014F2 decreases HCH cells apoptosis, the protein expression of Bax, Bcl-2, cleaved caspase-9 and -3 and cleaved PARP were determined. As shown in Figure 4, the expression of cleaved caspase-9 and -3 and cleaved PARP increased, the Bax/Bcl-2 ratio also increased, in IL-1β-stimulated HCH cells compared with the control group. These results were reversed in the range of 81.96–89.53% for Bax/Bcl-2 (*P* < 0.01), 47.82–94.14% for cleaved caspase-9 (*P* < 0.01), 26.49–86.17% for cleaved caspase-3 (*P* < 0.01) and 25.42–60.47% for cleaved PARP (*P* < 0.01) following the administration of LI73014F2 at concentrations of 12.5, 25 and 50 μg/mL.

### 3.6. Effects of LI73014F2 on the NF-κB p65 and p38 MAPK Signaling Pathway in IL-1β-stimulated HCH Cells

**Figure 5:**
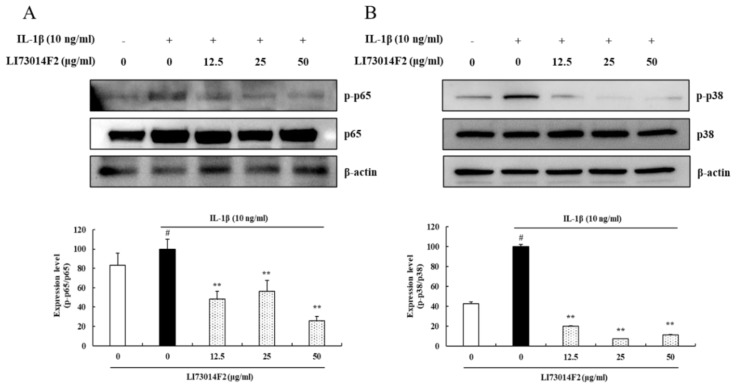
Effects of LI73014F2 on the NF-κB and Mitogen-activated protein kinase (MAPK) signaling pathway in IL-1β-stimulated HCH cells. HCH cells were pretreated with LI73014F2 (12.5, 25 and 50 µg/mL) for 24 h, followed by incubation with IL-1β (10 ng/mL) for 30 min. (**A**) The protein expression levels of NF-κB p65 (65 kDa) and phospho-NF-κB p65 (65 kDa) were examined by western blot analysis. (**B**) The protein expression levels of p38 MAPK (40 kDa) and phospho-p38 MAPK (43 kDa) were determined by western blot analysis. western blot bands were analyzed using Image J software. β-actin was used as a control for normalization. # *P* < 0.05, compared with the vehicle control group. * *P* < 0.05 and ** *P* < 0.01, compared with the IL-1β-treated control group.

The effects of LI73014F2 on IL-1β-induced NF-κB and MAPK activation were detected by western blot analysis. As shown in Figure 5, IL-1β stimulation significantly phosphorylated NF-κB p65 and p38 MAPK. However, treatment with LI73014F2 inhibited this IL-1β-induced phosphorylation in HCH cells, in the range of 43.67–74.21% and 80.10–92.59%, respectively, without affecting total protein expression (*P* < 0.01).

## 4. Discussion

Osteoarthritis is common form of arthritis in the knee and is accompanied with various symptoms such as inflammation, pain, swelling and stiffness, etc. The pathology of OA includes inflammatory reactions and multiple related pathways such as chondrocytes apoptosis pathways and MMPs in ECM matrix reduction, resulting in cartilage degradation and bone remodeling. Therefore, patients of osteoarthritis often derive less benefit from single therapy and need a treatment acting on multiple targets [25,26].

LI73014F2, a synergistic composition comprising aqueous extract of *T. chebula* fruit, alcohol extract of *C. longa* rhizome and *B. serrata* extract in a ratio of 2:1:2, was reported to inhibit COX-2 and 5-LOX activity [24]. The present study investigated the effects of LI73014F2 on IL-1β-stimulated inflammation in HCH cells, focusing on apoptosis and the expression of MMPs, as well as a potential underlying mechanism involving the MAPK/NF-κB signaling pathway.

IL-1β is an important inflammatory factor in the synovial fluid and joints and leads to the production and accumulation of inflammatory cytokines in the chondrocytes, which subsequently trigger the production of additional inflammatory cytokines that induce the expression of COX-2, 5-LOX and matrix-degrading enzymes such as MMPs [27,28]. COX-2 converts arachidonic acid into PGE_2_, which together with COX-2, sensitize peripheral receptors and cause pain. 5-LOX produces LTB_4_, which promotes the production and release of proinflammatory cytokines from synovial membranes. A previous study revealed that LI73014F2 inhibited COX-2 and 5-LOX enzymatic activity [24]. We, therefore, further investigated the synergistic effect of LI73014F2 in IL-1β-induced HCH cells. In the current study, we demonstrated that LI73014F2 significantly inhibited the production of PGE_2_ and LTB_4_ compared with its individual extracts. These results indicated that LI73014F2 is a synergistic composition that inhibits the synthesis of PGE_2_ and LTB_4_ by influencing the enzymatic activity of COX-2 and 5-LOX. These results were the same as those confirmed in previous clinical studies [24].

In the present study, we observed that treatment of HCH cells with IL-1β induced PGE_2_ and LTB_4_ release as well as COX-2, mPGES-1 and 5-LOX expression. However, LI73014F2 significantly inhibited the IL-1β-stimulated expression of COX-2, mPGES-1, PGE_2_, 5-LOX and LTB_4_, suggesting that LI73014F2 may inhibit the synthesis of PGE_2_ and LTB_4_ by influencing the enzymatic activity of COX-2 and 5-LOX, respectively. It is widely accepted that the activation of inflammatory cytokines, such as IL-1β, TNFα and IL-6, plays critical roles in the progression of OA [29]. In this study, we showed that LI73014F2 diminished the IL-1β-induced inflammatory response and decreased the expression of IL-1β, TNFα and IL-6 in HCH cells. These results indicate that LI73014F2 has anti-inflammatory effects against OA.

MMPs are important regulators in the progression of OA. MMP-3 and MMP-13 degrade collagens, proteoglycans and other extracellular matrix macromolecules in the cartilage [30]. MMP-2 and MMP-9, which are gelatinases, are correlated with the development of OA. MMP-9 and MMP-13 may play an important role in angiogenesis in OA [31]. Thus, targeting MMPs represents a strategy for potential therapy of OA. In the current study, we demonstrated that LI73014F2 decreased the IL-1β-dependent upregulated expression of MMP-2, MMP-3, MMP-9 and MMP-13 in HCH cells.

IL-1β has been shown to induce chondrocyte apoptosis [32]; therefore, IL-1β-treated HCH cells were used as an in vitro model system for investigating the anti-apoptotic effect of LI73014F2 in the present study. As an anti-apoptotic protein, Bcl-2 inhibits the release of cytochrome c and blocks the activation of caspase-9. Bax, a Bcl-2-like protein, is present in the cytosol and is involved in the initiation of apoptosis [33,34]. Caspases are a group of intracellular cysteine protease enzymes that degrade essential cellular proteins, leading to controlled cell death. There are two types of caspase enzymes. Initiator caspases (caspase-2, -8, -9, and -10) are activated through the apoptosis signaling pathways, and subsequently activate effector caspases (caspase-3, -6 and -7), which activate downstream pathways that lead to apoptosis [35,36,37]. Caspase-3 is a crucial biomarker of apoptosis that also acts as an apoptotic executor. An imbalance of Bax and Bcl-2 can result in a release of cytochrome c that activates caspase-9, which leads to cleavage of caspase-3. PARP, a downstream target of caspase-3, is a nuclear enzyme that is involved in DNA repair under physiological conditions; however, extensive activation of PARP promotes cell death [38,39,40]. We found that treatment with LI73014F2 downregulated the expression levels of apoptosis-related proteins in IL-1β-stimulated HCH cells. Our findings indicate that LI73014F2 could inhibit chondrocyte apoptosis.

MAPKs include p38 MAPK, extracellular signal-regulated kinase p44/42 MAPK and c-Jun N-terminal kinase. The activation of p38 MAPK is associated with chondrocyte apoptosis and inhibition of p38 MAPK has been shown to exhibit protective effects on cartilage degradation in different animal models. IL-1β, the most important cytokine produced by chondrocytes, has been shown to activate the MAPK pathway [41,42,43]. In this study, we found that LI73014F2 inhibited phosphorylation of p38 MAPK in IL-1β-stimulated HCH cells. Our results suggested that LI73014F2 attenuates chondrocytes apoptosis by inhibiting p38 MAPK signaling.

The NF-κB signaling pathway consists of ubiquitously expressed transcription factors involved in cellular differentiation, inflammatory responses, cell proliferation and cell death. Previous studies have confirmed that the NF-κB pathway is required for the expression of inflammation-related genes, including MMP-3, MMP-13 and COX-2, in chondrocytes [11,44]. The NF-κB p65 subunit is phosphorylated and translocated into the nucleus, where it binds to DNA promoter regions and activates the transcription of target genes, particularly during inflammation. Therefore, targeting NF-κB p65 may decrease inflammation in OA [45]. We investigated whether NF-κB p65 participated in the anti-inflammatory effects of LI73014F2 in OA. In this study, we found that LI73014F2 markedly suppressed the phosphorylation of NF-κB p65 in IL-1β-stimulated chondrocytes. These results indicated that the anti-inflammatory effects of LI73014F2 on chondrocytes are likely realized through the NF-κB pathway.

In summary, these inflammatory mediators not only stimulates the production of cartilage-degrading proteases to induce ECM degradation and apoptosis, but also contributes to OA-associated pain pathways. In addition, it shows that these factors could be potential targets for new biologic therapies that could be used to prevent OA and various symptoms in the future [46,47,48,49]. For this reason, in this present study suggests that LI73014F2 can relief of OA pathology by attenuation of chondrocyte apoptosis and expression of MMPs in ECM through reducing the pro-inflammatory mediator and inflammatory cytokines, including a cellular signaling pathway.

## 5. Conclusions

In conclusion, the present study revealed that LI73014F2 reduced the IL-1β-induced expression of pro-inflammatory mediator, inflammatory cytokines, MMPs and apoptosis-related proteins through suppressing phosphorylation of NF-κB p65 and phosphorylation of p38 MAPK in chondrocytes. These findings from in vitro studies demonstrated the molecular mechanism of action of LI73014F2 at the cellular level. Moreover, it also provides significant insights that supports the therapeutic efficacy of LI73014F2 in OA management.
